# Association between TSH Values and GFR Levels in Euthyroid Cases with Metabolic Syndrome

**DOI:** 10.1155/2021/8891972

**Published:** 2021-05-26

**Authors:** H. Keskin, K. Cadirci, K. Gungor, T. Karaaslan, T. Usta, A. Ozkeskin, A. Musayeva, F. Yesildal, F. Isman, H. Y. Zengin

**Affiliations:** ^1^Department of Internal Medicine, Goztepe Training and Research Hospital, Istanbul Medeniyet University, Istanbul 34772, Turkey; ^2^Department of Internal Medicine, Erzurum Regional Training and Research Hospital, Erzurum, Turkey; ^3^Department of Endocrinology, Goztepe Training and Research Hospital, Istanbul Medeniyet University, Istanbul, Turkey; ^4^Department of Nephrology, Goztepe Training and Research Hospital, Istanbul Medeniyet University, Istanbul, Turkey; ^5^Department of Internal Medicine, National Center of Oncology Department of Epidemiology and Statistics of Malignant Tumors, Baku, Azerbaijan; ^6^Department of Medical Biochemistry, Goztepe Training and Research Hospital, Istanbul Medeniyet University, Istanbul, Turkey; ^7^Department of Biostatistics, Hacettepe University Faculty of Medicine, Ankara, Turkey

## Abstract

**Background:**

Metabolic syndrome (MetS) is associated with the risk of developing chronic kidney disease. Although the negative effects of high thyroid-stimulating hormone (TSH) values on glomerular filtration rate (GFR) levels have been known for years, the negative effects of increased TSH on GFR in euthyroid cases have been reported in recent years. This study was aimed at investigating the association between the effect of increased TSH values and estimated-GFR (eGFR) levels in euthyroid cases with MetS.

**Methods:**

For this hospital-based descriptive study, 191 MetS cases (123 females, 68 males) were evaluated. Those whose TSH was not within 0.5–4.5 uIU/mL, eGFR was <40 mL/min/1.73 m^2^, and/or reported any thyroid/kidney disease were excluded. Partial correlation coefficients were calculated to investigate the relationship between the eGFR values and several other numerical variables while controlling for age and BMI in addition to the adjusted gender effect. Thereafter, the multiple linear regression analysis with a stepwise variable selection approach was used to reveal the independent factors that could affect the logarithmically transformed eGFR.

**Results:**

The median age was 52 (19–65) years, the median eGFR was 94.3 (41.3–194) mL/min/1.73 m^2^, and the median TSH was 1.58 (0.50–4.50) uIU/mL in the whole group. Increased TSH even in the normal range was associated with eGFR after adjusting for age and body mass index (BMI), especially in females. The high age (*b* = −0.160, *p*=0.005), high BMI (*b* = −0.134, *p*=0.020), high TSH (*b* = −0.380, *p* < 0.01), and high uric acid (*b* = −0.348, *p* < 0.01) were found as significant predictors of the eGFR in MetS patients.

**Conclusion:**

Independent of age and BMI, elevated TSH even in the euthyroid range showed an association with the eGFR in female MetS cases who had normal kidney functions. This correlation was stronger than the correlations between the eGFR and the MetS diagnostic parameters. These findings need further studies on the issue..

## 1. Introduction

Although the pathogenesis is not yet clearly understood, metabolic syndrome (MetS) is a collection of metabolic problems in which insulin resistance stands out as a main pathophysiological disorder. These patients are characterized by an increased risk of developing diabetes and cardiovascular disease. This syndrome is an important cause of morbidity and mortality, which tends to increase rapidly in both childhood and adult age groups, especially in developed and developing countries. According to the International Diabetes Federation data, it is estimated that 20–25% of the adult world population have MetS [1]. The METSAR study reported that the prevalence of MetS was 39.6% in females and 28% in males in Turkey [2].

Thyroid hormones are regulating hormones of the metabolism, and play a vital role in the progression of many important metabolic processes in the body. In numerous studies, the effect of change in thyroid function on renal function has been shown in hypo- and hyperthyroid patients. These hormones are important for the kidney to grow sufficiently and to complete its functional development, as well as maintenance of glomerular function [3, 4]. Also, it has shown that changes in thyroid functions are effective on the MetS frequency and MetS components [5–7]. Furthermore, significant positive correlations have been reported between the increase in thyroid-stimulating hormone (TSH) and the increase in the incidence of MetS and MetS components, even in euthyroid cases without subclinical hypo/hyperthyroidism [8]. Especially in patients with type 2 diabetes, even if TSH is in the euthyroid range, a significant decrease in GFR has been demonstrated with the increase in TSH levels [9]. Additionally, it has been reported that the incidence of diabetic nephropathy increases with this increase in TSH values [10]. Glomerular functions may be affected by several underlying mechanisms in autoimmune thyroiditis [11].

In this study, we evaluated the possible associations between TSH values and estimated-GFR (eGFR) levels in MetS patients whose TSH values were within the euthyroid range, and whose eGFR values were higher than 40 mL/min/1.73 m^2^.

## 2. Materials and Methods

The patients for this hospital-based descriptive study were chosen from the Internal Medicine Outpatient Clinics within a year. The data of all patients who were admitted to the clinics and who were estimated to meet MetS criteria were recorded. All of the participating patients were aged 18–65 years old (male and female). The physical examinations and body measurements were done by the same physician. Physical examination data, demographic features, and routine laboratory examination results were recorded. Patients who did not meet the criteria for MetS, whose eGFR level was lower than 40 ml/min/1.73 m^2^, whose hemoglobin level was lower than 11.0 gr/dl, whose TSH level was not in the euthyroid range (0.5–4.5 uIU/mL), who had a history of thyroid disease, who took any medication for thyroid disease, or who took contraceptive glucocorticoid, and/or antiepileptic drugs were excluded from the study. After using the exclusion criteria, a total of 193 patients (124 females, 69 males) from 246 patients (161 females, 85 males) were included in the study. Informed consent forms were obtained from all patients. Then, two cases that had outlier results were excluded from the study in the statistical analysis phase. Finally, the data of 191 cases (123 females, 68 males) were evaluated. The study was approved by the hospital's local ethics committee (2020/0427).

NCEP-ATP III consensus criteria were used as diagnostic criteria for metabolic syndrome [12]. In this study, estimated-GFR (eGFR) was used as an indicator of renal function since it is an easily calculated renal function indicator. The Modification of Renal Disease (MDRD) formula was used to calculate eGFR values. Homeostatic model assessment-insulin resistance (HOMA-IR) values were calculated according to the formula: fasting insulin (mIU/mL) × fasting glucose (mg/dL)/405. Body mass index (BMI) values were calculated according to the formula: weight (kg)/height (m^2^). All blood samples were taken after ≥12 hours of fasting. All laboratory examinations were performed in the same laboratory. TSH values were estimated by microparticle chemiluminescence immunoassay technology; this is a fully automated immunoassay analyzer from Abbott Architect i2000SR. The reference range for TSH was 0.35–4.94 mIU/L in the laboratory.

### 2.1. Statistical Analysis

To compare the gender subgroups in terms of numerical variables, the normality was assessed by the Kolmogorov–Smirnov normality test and the homogeneity of group variances was assessed by the Levene test. Independent samples *t*-test was used when the parametric test assumptions (assumption of normality and homogeneity of variances) were satisfied and means and standard deviations were used to describe those variables. Otherwise, the Mann–Whitney *U* test was used to assess the difference in gender groups with median (minimum-maximum values) as descriptive statistics due to the skewed nature of those variables. Frequencies and percentages were presented to describe the categorical variables. The association between gender and categorical variables was assessed with univariate analysis by the Pearson Chi-square test.

After evaluating the gender differences, partial correlation coefficients were calculated to investigate the relationship between the eGFR values and several other numerical variables while controlling for age and BMI in addition to the adjusted gender effect. In the literature, there are several approaches for transforming non-normally distributed numerical variables to more symmetrical structures such as Box-Cox transformations (Box GE and Cox D R. An Analysis of Transformations. Journal of the Royal Statistical Society. 1964, 211–252). When *λ* = 0, Box-Cox transformation is equal to logarithmic transformation. Therefore, we used Box-Cox transformation with *λ* = 0 for eGFR values, i.e., logarithmic transformation to obtain more symmetrical dependent variable values. Thereafter, the multiple linear regression analysis with a stepwise variable selection approach was used to reveal the independent factors that could affect the logarithmically transformed eGFR. The IBM SPSS Statistics (version 25.0 for Windows, Chicago, IL, USA) was used, and *p* < 0.05 was considered significant for all statistical analyses.

## 3. Results

There was no significant difference between male and female subgroups in terms of age (*p*=0.391). The majority of patients were on medication for type 2 diabetes and hypertension. Also, 38.1% of them were taking the antihyperlipidemic drug (s), and 19.4% of them were taking the drug (s) for cardiovascular diseases. Looking at the gender subgroups, there were not any significant differences in medication for type 2 diabetes (*p*=0.945), hyperlipidemia (*p*=0.870), and cardiovascular disease (*p*=0.406), but taking anti-hypertension drugs was higher in females (*p*=0.031). The mean-waist circumference value was higher in the male group as expected (*p*=0.033), while the mean-BMI was higher in women (*p* < 0.001) (Table 1).

HDL values were higher in the female group (*p* < 0.001) while there was no significant difference between male and female groups in terms of the other lipid parameters. Although all cases were euthyroid, TSH values were higher in females (*p*=0.031), too. However, free-T3 and free-T4 were similar in both gender groups. The hemoglobin, uric acid (UA), and serum creatinine values were significantly higher in the male group. The eGFR values were higher in females (*p* ≤ 0.001) (Table 2).

When we look at the whole group, there are weak/moderate negative correlations between the eGFR values and age, waist circumference, triglyceride, hemoglobin, microalbumin in spot urine, TSH values, and UA. There was also a weak positive correlation between the eGFR and HDL value in the whole group. However, there was not any statistically significant correlation between the eGFR and the BMI, blood pressure, fasting blood glucose, HbA1c, HOMA-IR, free-T3, free-T4, thyroid autoantibodies, total cholesterol, and LDL in the whole group (Table 3).

Since some of these tested variables may vary according to gender, and also some of these variations were statistically significant between the gender subgroup, all variables were examined in the gender subgroups as well as in the whole group. While some of the correlations detected in the main group disappeared in the gender subgroups, the others became less or more pronounced. For example, the correlations with age in the whole group were maintained in both genders while the correlations between the waist circumference, hemoglobin, and eGFR in the whole group disappeared in both genders. The correlations between the eGFR and TSH, the eGFR and UA were maintained in females while the correlations between eGFR and triglyceride, the eGFR, and microalbumin in spot urine were maintained in males only. On the other hand, there was no correlation between GFR and free-T3 in the whole group and female subgroup, while there was a moderate negative correlation between them in the male subgroup (Table 3).

Some variables showing significant correlations in partial correlation analysis such as age, BMI, waist circumference, HDL-cholesterol, triglyceride, microalbumin in spot urine, hemoglobin, TSH, and UA were also analyzed by stepwise multiple linear regression modeling. This modeling was conducted to determine the relationship between eGFR and various potential predictors. Based on this modeling, the age (*b* = −0.160, *p*=0.005), BMI (*b* = −0.134, *p*=0.020), TSH levels (*b* = −0.380, *p* < 0.01), UA levels (*b* = −0.348, *p* < 0.01) were found as significant predictors of the eGFR in MetS patients (Table 4).

## 4. Discussion

This hospital-based descriptive study aimed to investigate the relationship between eGFR and TSH in patients with euthyroid MetS. A correlation between the TSH and the eGFR values was detected in the female subgroup (*r* = −0.55; *p* < 0.001). The important thing is that this correlation was stronger than the correlations between the eGFR and the MetS parameters such as waist circumference, blood pressure, fasting blood glucose, HDL, and TG even after the adjustments for age and BMI in the female patients with MetS. Also, TSH was one of the most powerful predictors in the whole group and the female subgroup.

Garduno-Garcia et al., [13] in a study of 3,148 people, reported that increased TSH even in the euthyroid ranges caused a significant increase in the frequency of MetS even after excluding subclinical hypothyroidism. In the subgroup analysis of this large cohort study, it was stated that “free-T4, rather than TSH, is associated with risk of MetS and MetS components even in euthyroid subjects without subclinical hypothyroidism.” They also showed that levothyroxine (LT4) treatment has protective effects on kidney functions in patients with subclinical hypothyroidism [13]. However, in our study including 191 patients with euthyroid MetS, we could not detect any significant correlation or association between the free-T3, free-T4, and the eGFR levels in the whole group and the female subgroup, but there was a negative correlation between the eGFR and the free-T3 in the male subgroup as well as the moderate correlations between the eGFR and the TSH in the whole group and both gender subgroups. We also could not detect any significant correlation and relationship between the thyroid antibodies and eGFR. These results suggest that the relationship between TSH and eGFR did not arise from underlying undetected autoimmune thyroiditis cases in our study group. It is important to note that this high correlation and relationship between the TSH and the eGFR was independent of the underlying undetected autoimmune thyroiditis because the autoimmune disease may be related to glomerular dysfunction with several mechanisms [11].

Previous studies have reported that TSH levels increase with age; due to this, TSH levels can be followed up to (as high as) 8 uIU/mL without treatment in the geriatric population [14–16]. The decrease in eGFR with increasing age is considered to be a part of the natural aging process [17]. From this perspective, patients over 65 years old were not included in our study, and the correlation between the TSH and the eGFR was adjusted according to age. Even after these arrangements, the correlation between the TSH and the eGFR was detected stronger than the correlation between the age and the eGFR in our patients with MetS whose TSH level was distributed within the euthyroid range. In addition, independent of age, this correlation between the TSH and the eGFR was stronger than the correlations between the eGFR and the MetS parameters in the female patients with MetS.

Even though TSH level is in the euthyroid range, the positive correlation between TSH and BMI has been shown in many clinical studies [8, 18, 19]. It is known that the increase in BMI has negative effects on eGFR by several mechanisms [20]. Also, it was reported that MetS incidence increased 1.7–2-fold due to increased TSH; however, this increase is within the normal limits [18, 19]. In our euthyroid MetS patient groups, there was no significant correlation between the BMI and the eGFR in excluding for a weak correlation in the female subgroup (*r* = −0.22, *p*=0.015). This may be due to the fact that all our patients were diagnosed with MetS according to NCEP-ATP III consensus criteria, unlike the previous studies. During our literature surveys, we could not find enough comprehensive studies examining the relationship between TSH and eGFR in euthyroid MetS patients whose eGFR is >40 ml/min/1.73 m^2^.

Obesity and MetS are associated with the risk of developing microalbuminuria and chronic renal failure independent of diabetes and hypertension [21]. Contrary to what we expected, in our study, the negative effect of increased BMI on the eGFR was not significant in the whole group; however, there was a weak negative effect of increased BMI on the eGFR in the female subgroup only. Besides, the negative effect of increased waist circumference on the eGFR was significant in the whole group, but not in both gender subgroups. Waist circumference is an indicator of central obesity, and it is also among the diagnostic criteria of MetS. These results could remind us again that central obesity is more important than the other obesity style to affect the eGFR in patients with MetS. It was noteworthy that the correlation between TSH and eGFR was stronger than the correlation between waist circumference which is an indicator of central obesity and eGFR in the female subgroup.

The main pathophysiological element of MetS is insulin resistance; also it has been shown in several studies that insulin resistance is an independent risk factor for the development of chronic renal failure [22]. Thyroid hormones have a positive effect on insulin sensitivity, regulate renal blood flow, and affect eGFR by making intrinsic renal effects other than these pre-renal effects [23, 24]. In our study, there was no correlation between the eGFR levels and the fasting blood glucose, HbA1c, and HOMA-IR values. As it is known, renal dysfunction observed in MetS is the result of a chronic process. Our patients (66%) were taking various anti-diabetic drugs and these drugs are known to have positive effects on blood sugar regulation during the examination period. The lack of any correlation between the fasting blood glucose, HbA1c, HOMA-IR, and the eGFR in all groups in our study may be due to the efficacy of these anti-diabetic drugs at that time.

A negative correlation between increased blood pressure and eGFR is known [25]. However, we could not detect any significant correlation between eGFR and blood pressure. This may be the result of the effect of medical treatments; most of our patients are taking antihypertensive drugs (56% of the whole group) and we also only evaluated the blood pressure measurement during the examination, not before or after. On the other hand, the blood pressure levels detected during the examination may not reflect the patients' past blood pressure levels. Some similar hypotheses could have been valid for the lipid parameters due to the fact that 38.1% of the study participants were taking antihyperlipidemic drugs, but the increased HDL had positive correlations in the whole group and the female subgroup while the increased TG had a negative correlation with the eGFR in the whole group and both gender subgroups. Hereby, we have once again observed the effect of lipid parameters on renal functions.

The interaction between serum UA level and renal function has been investigated in many studies. Several studies reported a negative correlation between high serum UA level and renal function, but some studies did not report this correlation [26]. However, it is generally accepted that high serum levels of UA, which is also an inflammatory marker, have negative effects on renal function, hypertension, MetS, and CVD development through several pathways including endothelial dysfunction [26, 27]. The effect of high serum UA level on new-onset chronic kidney disease in individuals with normal renal function (eGFR >40 and/or >60 ml/min/1.73 m^2^) has been demonstrated by both studies and meta-analyses [26, 28]. And, this effect of UA has been independent of age, gender, BMI, HT, hypertriglyceridemia, type 2 diabetes, and MetS. Similar to the previous studies, there was a negative correlation between serum UA level and eGFR in our patient group with MetS whose eGFR was over 40 ml/min/1.73 m^2^, in our study as well.

The negative effect of high and low hemoglobin levels on eGFR has been known for many years (U-shape) [29]. However, we detected only the negative correlation between the Hb values and the eGFR levels. The reason for this result may be due to the fact that no upper limit for Hb was assigned in our study while those with Hb values less than 11 were excluded. As a result, we were able to see only a part of the U-shape.

In addition to our study, the results in a small patient group with MetS, considering the previous large population-based studies results, showed a negative correlation between TSH and eGFR in euthyroid subjects [30]; there can be a foresight that the upper limit of TSH may be kept around 2.5 rather than normal ranges in patients with euthyroid MetS. In normal population screening; it is recommended that the upper limit of TSH is 2.5 in healthy individuals without thyroid disease, and some attention should be paid to those between 2.5–4 uIU/mL [31]. It is well known that MetS is defined as a very important risk factor for the development of chronic renal failure, and its prevalence is increasing day by day. Maybe, the upper limit of TSH in MetS can be considered to be 2.5 uIU/mL, and cases whose TSH values are more than 2.5 uIU/mL cases should be examined in detail. Perhaps, in pregnant women, thyroid replacement therapy may be applied in these cases. Most importantly, in our study results, the negative correlation between the eGFR and elevated TSH (even in the euthyroid range) was stronger than the negative correlations between the eGFR and the MetS diagnostic parameters in the females. This negative correlation between the TSH and eGFR was independent of age and BMI. Also, in the female subgroup, this correlation was stronger than the correlation between eGFR and UA which is known as a systemic inflammatory marker. Two major limitations of the study were the single-center and small study group. However, they were also advantages in terms of data quality, physical examinations were performed by the same doctor, all laboratory examinations were performed in the same laboratory, and all patients' data were recorded by one person.

The association and relationship between increased TSH values even in the euthyroid range and eGFR levels in MetS cases are still not clear. In fact, renal dysfunction is associated with multiple alterations of thyroid hormone metabolism, including elevated basal TSH values, changes in TSH diurnal rhythm, altered TSH glycosylation, and impaired TSH renal clearance. More comprehensive, multicentric, and large studies should be conducted in patients with MetS to determine ideal TSH values for MetS in Turkey's population. Our results support that there is a significant relationship between TSH values and eGFR levels in our euthyroid Turkish cases with MetS after correcting for possible confounding factors.

## Figures and Tables

**Table 1:** Demographic characteristics of all cases with metabolic syndrome (MetS).

	All cases (*n* : 191)	Female (*n* : 123)	Male (*n* : 68)	*p*
	*n* (%)			
*Received treatment* (s) (yes) *for*				
(1) Type 2 diabetes	127(66.5)	82(66.7)	45(66.6)	0.945
(2) Hypertension	107(56.0)	76(61.8)	31(45.6)	**0.031**
(3) Hyperlipidemia	72(38.1)	47(38.5)	25(36.8)	0.870
(4) Cardiovascular disease	37(19.4)	26(21.1)	11(16.2)	0.406

	Median (min.-max.) or Mean ± standard deviation			
Age (year)	52 (19–65)	52 (19–65)	51.5 (27–65)	0.395
BMI (kg/m^2^)	31.2 (20.4–54.5)	32.2 (20.4–54.5)	29.7 (22.9–39.6)	**<0.001**
Waist circumference (cm)	101 (78–137)	100 (78–137)	103 (83–130)	**0.019**

*Blood pressure* (mmHg)				
(1) Systolic	149.1 ± 22.3	149.1 ± 23.2	148.9 ± 20.8	0.931^*∗*^
(2) Diastolic	82 (50–140)	82 (50–140)	82 (50–120)	0.890

BMI: body mass index, *p* < 0.05 was considered significant.^*∗*^Student's *t*-test was used.

**Table 2:** Laboratory parameters of all cases with metabolic syndrome (MetS).

	Median (min.-max.)			
	All cases (*n* : 191)	Female (*n* : 123)	Male (*n* : 68)	*p*
Fasting blood glucose (mg/dL)	127 (42.0–394)	127 (42–394)	129 (62–384)	0.756
HbA1c (%)	7 (5.20–12.7)	6.9 (5.4–12.7)	7 (5.2–12.4)	0.750
HOMA-IR	2.64 (0.64–20.2)	2.45 (0.64–20.2)	3.18 (0.77–10.6)	0.242
Total cholesterol (mg/dL)	192 (103–347)	191 (103–342)	193 (118–347)	0.604
HDL-cholesterol (mg/dL)	45 (22–96)	48 (27–96)	42 (22–82)	**<0.001**
LDL-cholesterol (mg/dL)	114 (27–400)	114 (27–400)	113 (43–215)	0.639
Triglyceride (mg/dL)	145 (45–1245)	144 (47–451)	144 (45–1245)	0.812
TSH (uIU/mL)	1.58 (0.50–4.50)	1.65 (0.57–4.5)	1.37(0.5–4.5)	**0.031**
Free-T3 (pg/mL)	3.11 (0.07–4.02)	3.07 (0.07–4.02)	3.17 (2.35–3.86)	0.109
Free-T4 (ng/mL)	0.87 (0.46–3.40)	0.87 (0.46–3.4)	0.85 (0.62–1.37)	0.240
Anti-TPO (IU/mL)	0.6 (0.10–170)	0.7 (0.1–170)	0.6 (0.2–86)	0.236
Anti-TG (IU/mL)	0.9 (0.90–722)	0.9 (0.9–456)	0.9 (0.9–722)	**0.042**
Hemoglobin (g/dL)	13.8 (11.0–17.8)	12.8 (11–15.6)	14.9 (11–17.8)	**<0.001**
Uric acid (mg/dL)	4.8 (1.80–9.30)	4.45 (1.8–9.3)	5.2 (2.8–8.6)	**0.004**
eGFR (mL/min/1.73 m^2^)	94.3 (41.3–194)	101 (46–193)	78 (41–153)	**<0.001**
Serum creatinine (mmol/L)	0.76 (0.38–1.50)	0.69 (0.38–1.25)	0.91 (0.62–1.50)	**<0.001**
Microalbumin in spot urine (mg/L)	1.21 (0.09–230)	1.21 (0.09–179)	1.27(0.30–230)	0.798

HOMA-IR: the homeostatic model assessment-insulin resistance, eGFR: estimated glomerular filtration rate. *p* < 0.05 was considered significant.

**Table 3:** The partial correlation coefficients between the estimated-GFR (log_10_-eGFR) and the other parameters in the whole group and the gender subgroups.

	All cases (*n* = 191)		Female (*n* = 123)		Male (*n* = 68)	
	*r*	*p*	*r*	*p*	*r*	*p*
Age (year)^1^	−0.27	**<0.001**	−0.32	**<0.001**	−0.36	**0.003**
BMI (kg/m^2^)^2^	−0.04	0.583	−0.22	**0.015**	−0.07	0.548
Waist circumference (cm)^3^	−0.33	**<0.001**	−0.13	0.176	−0.13	0.282
*Blood pressure* (mmHg)^3^						
(1) Systolic	+0.01	0.863	+0.05	0.558	−0.08	0.549
(2) Diastolic	−0.03	0.700	+0.05	0.556	−0.17	0.168
Fasting blood glucose (mg/dL)^3^	+0.09	0.378	+0.08	0.516	+0.24	0.155
HbA1c (%)^3^	+0.10	0.288	+0.10	0.409	+0.22	0.188
HOMA-IR^3^	−0.04	0.655	+0.01	0.935	+0.14	0.407
Total cholesterol (mg/dL)^3^	+0.01	0.895	−0.02	0.792	−0.05	0.715
HDL-cholesterol (mg/dL)^3^	+0.23	**0.002**	+0.09	0.331	+0.22	0.075
LDL-cholesterol (mg/dL)^3^	+0.04	0.562	+0.01	0.962	+0.14	0.272
Triglyceride (mg/dL)^3^	−0.19	**0.009**	−0.89	0.336	−0.25	**0.044**
Microalbumin in spot urine (mg/L)^3^	−0.23	**0.035**	−0.17	0.240	−0.48	**0.009**
Hemoglobin (gr/dL)^3^	−0.25	**0.023**	−0.01	0.932	+0.31	0.106
TSH (uIU/mL)^3^	−0.29	**0.009**	−0.55	**<0.001**	−0.30	0.119
Free-T3 (pg/mL)^3^	+0.16	0.159	+0.23	0.104	+0.44	**0.020**
Free-T4 (ng/dL)^3^	+0.17	0.130	+0.11	0.427	+0.17	0.392
Anti-TPO (IU/mL)^3^	−0.05	0.629	−0.14	0.341	−0.03	0.876
Anti-Tg (IU/mL)^3^	+0.08	0.452	−0.01	0.992	−0.04	0.854
Uric acid (mg/dL)^3^	−0.40	**<0.001**	−0.40	**0.004**	−0.19	0.331

^1^Adjusted for BMI, ^2^adjusted for age, ^3^adjusted for age and BMI, *r*: the partial correlation coefficient, *p* < 0.05 was considered significant.

**Table 4:** Multiple linear regression model with estimated-GFR (log_10_-eGFR).

		Whole group (*n* = 191)		
Dependent variable	Independent predictors	*b*	*t*	*p*
Estimated-GFR	Age	−0.160	−2.818	0.005
	BMI	−0.134	−2.354	0.020
	Gender	−0.406	−6.957	<0.001
	TSH	−0.380	−6.760	<0.001
	Uric acid (mg/dL)	−0.348	−6.153	<0.001

*b*: standardized coefficients, *p* < 0.05 was considered significant.

## Data Availability

The datasets generated and/or analyzed during the current study are available from the corresponding author on reasonable request.

## References

[1] Albert S. G., Zimmet P. (2019). The IDF consensus worldwide definition of the metabolic syndrome. https://file:///C:/Users/hp/Downloads/IDF_Meta_def_final%20(1).pdf.

[2] Kozan O., Oguz A., Abaci A. (2007). Prevalence of the metabolic syndrome among Turkish adults. *European Journal of Clinical Nutrition*.

[3] Wang K., Xie K., Gu L. (2018). Association of thyroid function with the estimated glomerular filtration rate in a large Chinese euthyroid population. *Kidney and Blood Pressure Research*.

[4] Mariani L. H., Berns J. S. (2012). The renal manifestations of thyroid disease. *Journal of the American Society of Nephrology*.

[5] Kumar H. K., Yadav R. K., Prajapati J., Reddy C. V., Raghunath M., Modi K. D. (2009). Association between thyroid hormones, insulin resistance, and metabolic syndrome. *Saudi Medical Journal*.

[6] Delitala A. P., Fanciulli G., Pes G. M., Maioli M., Delitala G. (2017). Thyroid hormones, metabolic syndrome and its components. *Endocrine, Metabolic & Immune Disorders - Drug Targets*.

[7] Jang J., Kim Y., Shin J., Lee S. A., Choi Y., Park E. C. (2018). Association between thyroid hormones and the components of metabolic syndrome. *BMC Endocrine Disorders*.

[8] Chang Y.-C., Hua S.-C., Chang C.-H. (2019). High TSH level within normal range is associated with obesity, dyslipidemia, hypertension, inflammation, hypercoagulability, and the metabolic syndrome: a novel cardiometabolic marker. *Journal of Clinical Medicine*.

[9] Zhang Y., Wang Y., Tao X. J. (2018). Relationship between thyroid function and kidney function in patients with type 2 diabetes. *International Journal of Endocrinology*.

[10] Wang J., Li H., Tan M. (2019). Association between thyroid function and diabetic nephropathy in euthyroid subjects with type 2 diabetes mellitus: a cross-sectional study in China. *Oncotarget*.

[11] Santoro D., Vadalà C., Siligato R., Buemi M., Benvenga S. (2017). Autoimmune thyroiditis and glomerulopathies. *Frontiers in Endocrinology (Lausanne)*.

[12] National Cholesterol Education Program (NCEP) Expert Panel on Detection, Evaluation, and Treatment of High Blood Cholesterol in Adults (Adult Treatment Panel III) (2002). Third report of the national cholesterol education program (NCEP) expert panel on detection, evaluation, and treatment of high blood cholesterol in adults (adult treatment panel III) final report. *Circulation*.

[13] Garduño-Garcia J. d. J., Alvirde-Garcia U., López-Carrasco G. (2010). TSH and free thyroxine concentrations are associated with differing metabolic markers in euthyroid subjects. *European Journal of Endocrinology*.

[14] Surks M. I., Hollowell J. G. (2007). Age-specific distribution of serum thyrotropin and antithyroid antibodies in the U.S. population: implications for the prevalence of subclinical hypothyroidism. *The Journal of Clinical Endocrinology & Metabolism*.

[15] Waring A. C., Rodondi N., Harrison S. (2012). Thyroid function and prevalent and incident metabolic syndrome in older adults: the Health, Ageing and Body Composition Study. *Clinical Endocrinology*.

[16] Duntas L. H., Yen P. M. (2019). Diagnosis and treatment of hypothyroidism in the elderly. *Endocrine*.

[17] Biondi B. (2010). Thyroid and obesity: an intriguing relationship. *The Journal of Clinical Endocrinology & Metabolism*.

[18] Oh J.-Y., Sung Y.-A., Lee H. J. (2013). Elevated thyroid stimulating hormone levels are associated with metabolic syndrome in euthyroid young women. *The Korean Journal of Internal Medicine*.

[19] Ruhla S., Weickert M. O., Arafat A. M. (2010). A high normal TSH is associated with the metabolic syndrome. *Clinical Endocrinology*.

[20] Chang A. R., Surapaneni A., Kirchner H. L. (2018). Metabolically healthy obesity and risk of kidney function decline. *Obesity*.

[21] Weisinger J. R., Kempson R. L., Eldridge F. L., Swenson R. S. (1974). The nephrotic syndrome: a complication of massive obesity. *Annals of Internal Medicine*.

[22] Nerpin E., Risérus U., Ingelsson E. (2008). Insulin sensitivity measured with euglycemic clamp is independently associated with glomerular filtration rate in a community-based cohort. *Diabetes Care*.

[23] Mullur R., Liu Y.-Y., Brent G. A. (2014). Thyroid hormone regulation of metabolism. *Physiological Reviews*.

[24] Basu G., Mohapatra A. (2012). Interactions between thyroid disorders and kidney disease. *Indian Journal of Endocrinology and Metabolism*.

[25] Hsu C.-y., McCulloch C. E., Darbinian J. (2005). Elevated blood pressure and risk of end-stage renal disease in subjects without baseline kidney disease. *Archives of Internal Medicine*.

[26] Li L., Yang C., Zhao Y., Zeng X., Liu F., Fu P. (2014). Is hyperuricemia an independent risk factor for new-onset chronic kidney disease?: a systematic review and meta-analysis based on observational cohort studies. *BMC Nephrol*.

[27] Kang D.-H., Park S.-K., Lee I.-K., Johnson R. J. (2005). Uric acid-induced C-reactive protein expression: implication on cell proliferation and nitric oxide production of human vascular cells. *Journal of the American Society of Nephrology*.

[28] Jalal D. I., Chonchol M., Chen W., Targher G. (2013). Uric acid as a target of therapy in CKD. *American Journal of Kidney Diseases*.

[29] Isakov E., Froom P., Henig C., Barak M. (2014). Anemia and estimated glomerular filtration rates. *Annals of Clinical and Laboratory Science*.

[30] Åsvold B. O., Bjøro T., Vatten L. J. (2011). Association of thyroid function with estimated glomerular filtration rate in a population-based study: the HUNT study. *European Journal of Endocrinology*.

[31] Biondi B. (2013). The normal TSH reference range: what has changed in the last decade?. *The Journal of Clinical Endocrinology & Metabolism*.

